# Recurrence of portosystemic encephalopathy in cirrhotic patients and its risk factors

**DOI:** 10.12669/pjms.40.1.8025

**Published:** 2024

**Authors:** Masood Muhammad Karim, Abdullah Bin Khalid, Zahabia Sohail, Mohammad Yasrab

**Affiliations:** 1 Masood Muhammad Karim, Fellow, Aga Khan University Hospital, Karachi, Pakistan; 2 Abdullah Bin Khalid, Lecturer, Department of Medicine, Lecturer, Dow University of Health Science, Karachi, Pakistan, Aga Khan University Hospital, Karachi, Pakistan; 3 Zahabia Sohail, Resident, Post Graduate Medical Education, Aga Khan University Hospital, Karachi, Pakistan; 4 Mohammad Yasrab, Student, Undergraduate Medical Education, Aga Khan University, Karachi, Pakistan

**Keywords:** Portosystemic Encephalopathy, Risk Factors, Recurrence

## Abstract

**Objectives:**

Recurrent episodes of Portal Systemic Encephalopathy (PSE), poses a significant burden of illness on the patients and healthcare system. The objective of this study was to assess the recurrence of PSE in cirrhotic patients after index episode of PSE and to identify various risk factors associated with it.

**Methods:**

A retrospective, single-centre study was conducted at Aga Khan University Hospital over a span of one year. Patients who were admitted first time with PSE and admitted within three months of index PSE were enrolled in the study. Variables assessed were demographic data, associated comorbid conditions, aetiology of cirrhosis, Child-Turcotte-Pugh (CTP) score, Model of End-Stage Liver Disease (MELD) score, PSE grade, laboratory tests, ascites with spontaneous bacterial peritonitis (SBP), variceal bleeding. Statistical analysis was done and variables of those who developed recurrence were compared with those who did not.

**Results:**

Fifty one patients were recruited. Thirty three (64.7%) were readmitted with PSE. On comparative analysis of both groups; infection, Meld score, low albumin, and raised total bilirubin showed significant P-value (<0.05).

**Conclusion:**

Identification of risk factors during assessment can reduce the recurrence of PSE. We would recommend to validate result of our study on a large scale prospectively.

## INTRODUCTION

Portal systemic Encephalopathy (PSE) represents a reversible impairment of neuropsychiatric function associated with impaired hepatic function.^1^ It is a common complication of cirrhosis in advanced stages that carries a high psychosocial and financial burden of illness, hospitalisation and mortality. Cirrhosis mortality rose by 65% from 2008 to 2016 and is expected to triple by 2030,^2^ and amongst many other contributing causes, PSE is an important one. A study in Pakistan found that patients with cirrhosis who were admitted with hepatic encephalopathy (HE) had a nearly eight times greater mortality risk than those who did not.^3^

Recurrent PSE denotes bouts of encephalopathy that occur with a time interval of six months or less.^4^ The risk for the first bout of overt hepatic encephalopathy is 5%-25% within five years after cirrhosis diagnosis. Subjects with a previous bout of PSE were found to have a 40% cumulative risk of recurring PSE at one year and subjects with recurrent PSE have a 40% cumulative risk of another recurrence within six months, despite lactulose treatment.^5^

The exact underlying process by which hepatic encephalopathy develops is unknown, but high levels of substances produced by the digestive breakdown of proteins, such as ammonia, are believed to play a major role.^6^ Presence of other complications of cirrhosis such as variceal bleeding or ascites can also precipitate an episode of hepatic encephalopathy.

Recurrence of PSE in cirrhotic patients is associated with worse outcomes in term of disease progression and with deterioration in the quality of life. Each episode of PSE increases the likelihood of future episodes, resulting in poorer recovery and outcomes each time and unleashing a vicious cycle.^7^ After the first episode of PSE, the survival probability decreases to 42% at one year of follow-up and 23% at three years.^8^ Alterations in brain functioning produces cognitive impairment which results in utilization of more health care resources in adults than other manifestations of liver disease.^5^

The primary treatment of HE is the identification and treatment of the precipitating factors in order to prevent development of further episodes of PSE. The patient’s risk factors may allow for closer monitoring to preserve quality of life and to reduce healthcare burden. In a developing country like Pakistan with poor accessibility to adequate healthcare facilities and resources, prevention of recurrence is of paramount importance. Therefore, this study was carried out with the objective of ascertaining the recurrence of PSE in cirrhotic patients after the first episode of PSE and its risk factors.

## METHODS

This is a retrospective study which was done at The Aga Khan Hospital, Karachi during January 2019 till December 2019. All cirrhotic adult patients greater than 18 years of age with admitting diagnosis of index PSE were included in the study. Patients with hepatocellular carcinoma (HCC), metastatic liver, cerebrovascular accidents, and central nervous system diseases or with psychiatric illness were excluded from our study.

### Ethical Approval

The study was approved by hospital ethics committee on April 12^th^ 2022 Ref. 2022-7240-20965.

After enrolment in the study, charts of recruited patients were reviewed for clinical characteristics, the cause of PSE and clinical outcome. Records of recruited patient were reviewed for three months after index PSE whether they needed re-admission for recurrence. Diagnosis of hepatic encephalopathy was graded from I to IV according to the West Haven criteria.^9^

The questionnaire included variables such as age, gender, comorbidities, aetiology of cirrhosis, PSE grade, serum sodium, potassium, INR (International Normalized Ratio), creatinine, and albumin at the time of the index PSE admission. Severity of chronic liver disease in patients was categorized according to CTP classification and was scored as Child A, B or C. MELD score used to predict short term mortality risk in cirrhotic patients was also recorded and a score greater than 18 was considered as high.

All the data was then recorded in the statistical software, SPSS version 22, and descriptive analysis was performed. Variables of patients who developed recurrence of PSE and those who did not develop PSE were compared and statistical analysis was done. Unpaired t-tests were used for continuous variables and Chi-square test for categorical variables. A p-value of <0.05 was considered significant.

## RESULTS

A total number of 51 adults, diagnosed cases of chronic liver disease, who presented with hepatic encephalopathy irrespective of cause were included. It was found that there was almost equal representation from each gender in our population. Mean age of the patients was found to be 59±10 years (Table-I).

**Table-I:** Patient demographics at index PSE admission.

Variables	Frequency (Percentage)
Age (Years)	59+/-10 (Mean)
***Gender***	
Male	29 (57%)
Female	22 (43%)
***Comorbids***	
Hypertension	24 (47%)
Diabetes	20 (39%)
Other	7 (14%)
***Etiology***	
HCV	30 (59%)
HBV	14 (27%)
Alcoholic	03 (6%)
Other	04 (8%)
***PSE Grade***	
2	4 (7%)
3	17 (33.5%)
4	30 (59.5%)
***CTP Score***	
CTP-A	0 (0%)
CTP-B	13 (24%)
CTP-C	38 (76%)

The most common cause of chronic liver disease was found to be HCV in our patients (59%). On index admission, majority of our patients presented with Grade-4 encephalopathy (59.5%) and CTP C CLD (76%) (Table-II).

**Table-II:** Continued. Patient demographics at index PSE admission.

Variables	Frequency (Percentage)
***MELD score***	
<18	18 (35%)
>18	33 (65%)
***Total Bilirubin (mg/dL)***	
<3	25 (50%)
>3-6	21 (41.2%)
>6	5 (9.8%)
***Albumin (g/dL)***	
>3.5,	21(41.2%)
2.8-3.5	20 (39.2%)
<2.8	10 (19.6%)
Urinary tract Infection (UTI)	10 (19.4%)
Acute kidney Injury (AKI)	8 (15.6%)
Constipation	8 (15.6%)
Hyponatremia	9 (18%)
Hypokalemia	7 (14.4%)
SBP	6 (11%)
Variceal bleeding	2 (6%)

Majority of the patients at index admission had MELD scores >18 (65%). Nearly half of the patients had Total bilirubin (TB) levels of <3 and around 90% had a TB of less than six. Other parameters that were recorded were the presence of UTI, hypokalaemia, hyponatremia, AKI, constipation, SBP and variceal bleeding upon index admission.

All 51 patients were followed for three months, to see if they developed recurrence of PSE and it was found that almost 2/3^rd^ of the patients got readmitted with another episode of PSE. Those who developed recurrence of PSE and those who did not were divided into two groups and their variables were compared. Mean age of the patients was essentially same in both the groups. Laboratory parameters of serum creatinine, serum sodium and serum potassium levels had no statistically significant difference between the two groups (p-value of > 0.05). However, patients who developed infection, including SBP and UTI, were almost four times as much in recurrent group (n= 20, 60%) than non-recurrent group (n= 4, 22%) with statistically significant P-value of 0.01. Amongst the recurrent group (n=33), Infection was present in nearly two-thirds (n=20, 60%) of the patients. Patients who developed recurrence of PSE had a high MELD score of 19 +/- 2 whereas patients who did not develop PSE recurrence had mean MELD score of 14. This was found to be statistically significant with P value of 0.03. Similarly, low serum albumin (mean 2.2) and high bilirubin levels (mean 4.5) in the recurrent group were found to be statistically significant differentiating factors between the two groups (Table-III).

**Table-III:** Multivariate analysis of continuous and categorical variables between recurrent and non-recurrent PSE groups.

Variables	Recurrent PSE (n=33)	Non-Recurrent PSE (n=18)	P Value (Significance= <0.05)
Age (years)	57 ± 9	55 ± 6	0.85
Infection^1^	20 (60%)	4 (22%)	0.01
MELD Score	19 ± 2	14	0.03
INR	1.5 ± 0.5	1.4 ± 0.7	0.90
Albumin (g/dL)	2.2 ± 0.8	2.9 ± 0.5	0.04
Creatinine(mg/dL)	1.2 ± 0.4	1.3 ± 0.3	0.83
Sodium (mEq/L)	130 ± 2	133 ± 2	0.73
Potassium(mEq/L)	3.8 ± 1	3.5 ± 0.5	0.82
CTP Score	11 ± 4	10 ± 2	0.90
Bilirubin(mg/dL)	4.5 ± 2	2 ± 1	0.04

1 SBP, UTI.

## DISCUSSION

Our study was conducted in order to uncover risk factors associated with recurrence of PSE amongst cirrhotic patients. A total of 51 patients were included in the study who developed index PSE. Of those, 33 developed recurrences of PSE within 90 days. Upon analysis, factors that significantly differed between the two groups included concomitant infection, a higher MELD score, lower serum albumin levels, and higher total bilirubin levels.

We found that over half (60%, p = 0.01) of PSE recurrence cases were associated with infection, most commonly urinary tract infections (UTIs) and spontaneous bacterial peritonitis (SBP). Studies have established that infections act as an independent risk factor in the development and precipitation of overt PSE. Furthermore, PSE itself increases the risk of subsequent infection as well, thus creating a feedback cycle where one predisposes to the other.^10^ Maqsood et al. found that nearly half (44%) of all occurrences of PSE were precipitated by infections, and this figure was as high as 67% in a study by Devrajani.^11^,^12^ Our study corroborates the significance of infection, and thus, index PSE patients who develop an infection should be considered at significant risk for recurrence.

Albumin, levels (2.2 ± 0.8 vs. 2.9 ± 0.5, p = 0.04) were also significantly lower in recurrences of PSE. One of the aforementioned studies in Pakistan also found that 59% of their PSE cases had hypoalbuminemia.^12^ Furthermore, we also saw that cases of PSE recurrence had total bilirubin levels of 4.5 ± 2 compared to 2 ± 1 (p = 0.04) in those who did not develop recurrence. Bilirubin is an important predictor of mortality in patients with PSE and a predictor of length of hospital stay.^13^ This is likely because hepatocyte damage increases conjugated bilirubin, and lower serum albumin increases unbound unconjugated bilirubin.

The MELD score was another significant variable in our study with a value of 19 ± 2 in cases of recurrence as opposed to 14 in the comparison group (p = 0.03). Bajaj et al. in their multivariate analysis revealed that the MELD score was an important predictor of recurrence.^14^ Hu et al noted in their study that INR at discharge (>1.62) independently predicted overall readmission after index PSE, and that the MELD score was the only variable that predicted readmission specifically because of PSE.^15^ INR has been associated with a longer hospital stay in PSE patients. However, it did not significantly differ in our particular study. This could be due to population differences and small sample size. Nonetheless, a lower MELD score at index PSE should raise concern about subsequent recurrences.

Other studies have shown different predictors for PSE and recurrences. Duah et al. also found in their study that infections were the most common precipitating factor of PSE as in our study, but their study also revealed that severe ascites, low platelet count, high creatinine, BUN, and CTP score were independent predictors as well.^16^ While these may be important predictors of index PSE, our study did not show significant differences in creatinine, BUN, and CTP score with regards to recurrence of PSE. There could be a number of reasons regarding the difference here. In our particular healthcare set up, in light of the inadequacy of healthcare services in an underdeveloped country like Pakistan, when patients do get admitted with index PSE, they are typically not discharged until their creatinine levels and electrolytes are under control. This might also explain why we did not find a significant association with hyponatremia and hypokalaemia. Regarding potassium levels, Gaduputi et al in their study showed that levels <4mEq/L were associated with longer hospital stays,^17^ and while mean potassium levels were below 4mEq/L in both of our study groups, they did not differ significantly between them.

It is important to recognize these predictors and address them because readmissions in decompensated cirrhosis pose to be a significant burden in both costs and resources, to patients, their caregivers and the healthcare system at large. Roggeri et al. in their study conducted in Italy found that 42.5% of patients with index PSE had at least one rehospitalization, doubling their healthcare costs,^18^ and PSE is amongst the most common reasons seen in multiple studies.^19^,^20^ Furthermore, recurrences after initial overt PSE are associated with poorer quality of life and persistence of cognitive deficits. Such trends are seen across healthcare setups and countries, emphasizing the need to find and address gaps.

### Limitations of the study

One of these is the possibility that there may have been patients who did get readmitted but to a different healthcare centre. As many one in every four readmissions are to another hospital^21^, and this fraction was one in three in a study conducted in the US by Okafor et al.^22^ It’s important to keep this in check in studies, and it is likely that many patients develop PSE but were never brought to our facility due to poor access and healthcare infrastructure. To further build upon the findings of this study, we suggest undertaking prospective and retrospective studies over a wider time period with larger sample sizes.

Due to the long-term nature of cirrhosis, it is imperative to continue post-discharge care effectively to reduce readmissions and recurrences of PSE. Risk factors should be optimized prior to discharge and effective counselling of the patient and attendants can go a long way, as shown by Shaw et al. who discussed that having a more integrated nutrition counselling and planning programme may result in better outcomes of hepatic encephalopathy.^23^ Rosenblatt et al. in their recent study covering strategies to prevent recurrences of PSE highlighted simpler measures such as telemedicine to improve medication adherence.^24^ Not all countries are developed enough to have a robust network of healthcare facilities and personnel working together, including ours, but these alternatives could certainly be explored.

## CONCLUSION

Portal systemic encephalopathy in itself is a huge burden and carries significant morbidity and mortality. The fact that there is documented evidence that index episode of PSE is followed by increasing risk of recurrence with each subsequent admission warrants our attentions. Through this study, certain risk factors have shown to influence this recurrence. MELD score >18, raised TB > 6mg/dl, decreased sodium, low albumin level and infections are significantly associated with PSE recurrence. Knowing these risk factors, we can tailor our limited resources towards such patients and maximize good outcomes. To draw the most benefit, these efforts should be contextualized and specific to the patient population and the local healthcare system.

### Authors’ Contributions:

**MK:** Concept, design, undertook the data analyses and critical revision of the manuscript.

**AK:** Study conception, critical revisions, and final approval of the manuscript.

**ZS:** Data collection, drafting the manuscript, edited and revised the manuscript

**MY:** Interpretation of data, study finalization, prepared and reviewed the manuscript.
